# Classifier Level Fusion of Accelerometer and sEMG Signals for Automatic Fitness Activity Diarization

**DOI:** 10.3390/s18092850

**Published:** 2018-08-29

**Authors:** Giorgio Biagetti, Paolo Crippa, Laura Falaschetti, Claudio Turchetti

**Affiliations:** DII—Dipartimento di Ingegneria dell’Informazione, Università Politecnica delle Marche, Via Brecce Bianche 12, I-60131 Ancona, Italy; g.biagetti@univpm.it (G.B.); l.falaschetti@univpm.it (L.F.); c.turchetti@univpm.it (C.T.)

**Keywords:** accelerometer, electromiography, sEMG, data fusion, activity diarization, wearable system, classifier level fusion

## Abstract

The human activity diarization using wearable technologies is one of the most important supporting techniques for ambient assisted living, sport and fitness activities, healthcare of elderly people. The activity diarization is performed in two steps: the acquisition of body signals and the classification of activities being performed. This paper presents a technique for data fusion at classifier level of accelerometer and sEMG signals acquired by using a low-cost wearable wireless system for monitoring the human activity when performing sport and fitness activities, as well as in healthcare applications. To demonstrate the capability of the system of diarizing the user’s activities, data recorded from a few subjects were used to train and test the automatic classifier for recognizing the type of exercise being performed.

## 1. Introduction

Techniques based on acceleration and surface electromyographic (sEMG) signals are two research branches in the field of human activity pattern recognition.

Acceleration-based techniques are well suited to distinguish noticeable and large scale gestures with different hand trajectories of forearm movements [1,2,3,4]. In particular, they have been demonstrated to be effective in classifying activities that involve repetitive body motions, such as walking, running, cycling, lifting weights, climbing stairs [5]. Additionally, nowadays acceleration-based techniques are easy to implement because inertial sensors are usually embedded in smartphones and smartwatches [6,7,8,9,10] that are widespread and very common in people playing sports. However with acceleration signals derived from such devices only, recognizing different activities or sport exercises with similar arm movements could be a difficult task.

To this end the electrical signals captured from muscles’ activity are very helpful in recognizing this kind of exercises as well as in monitoring person’s body posture, physical performance, and fitness level as it has been demonstrated by recent works [11,12,13,14,15,16]. This is due to the fact that sEMG signals can be collected using noninvasive sensor devices and they are relatively easy to acquire. Indeed, these signals are derived from the electrical potentials generated by muscle contractions, therefore they can be captured simply by contacting electrodes to the skin surface [17,18].

SEMG-based activity recognition techniques use multi-channel EMG signals which contain rich information about movements of various size scales. To this end a low-cost wireless system specifically designed to acquire fitness metrics from surface electromyographic (sEMG) and accelerometric signals has been adopted [19]. The system consists of three ultralight (23 g) wireless sensing nodes that acquire, amplify, digitize, and transmit the sEMG and accelerometer signals to one base station through a 2.4 GHz radio link using a custom-made communication protocol. The base station is connected via USB to a control PC running a user interface software for data analysis and storage.

The sensing nodes use a carefully designed high-input-impedance (20 MΩ differential) low-noise amplifier for detecting the low amplitude sEMG signal. It is then filtered to only let the useful 5 Hz to 500 Hz band through, taking care of rejecting the motion-induced artifacts at frequencies below 5 Hz before the amplifier gain stages could saturate, and then digitized. The amplifier also has a programmable gain stage to adapt the system for possible application to very differently sized body muscles, ranging from major limb motor muscles to facial expression muscles.

With the above considerations in mind, combining sEMG and accelerometer sensors in a single device allows also to obtain all the necessary information for accurately examining muscle activity, force, fatigue, directionality and acceleration that are of essential importance in sports performance evaluation, injury prevention, rehabilitation, and human activity monitoring in general [20,21,22,23,24,25,26,27].

Considering the complementary features of accelerometer and sEMG measurements, we believe that their combination by using classifier level fusion techniques will increase the number of discriminable arm exercises and accuracy of the recognition system.

There are several sensor fusion techniques, applied to the sensors embedded in smartphones, smartwaches, as a means to help identify the mobile device user’s daily activities or sport exercises. Sensor data fusion methods help to consolidate the signals collected from different body sensors, increasing the performance of the algorithms for the recognition of the different activities [28,29,30,31,32]. However, due to low memory, low battery life and low processing power constraints, some data fusion techniques are not suited to this scenario.

There are different ways of combining data from various types of sensors to help improve recognition. Data can be combined on a feature level, or at the classifier level, whereas base classifiers are built on the different types of data separately, and a so called a meta-level classifier then combines their outputs [33]. This latter approach is deemed to produce better results, and so is the one employed in this paper.

This paper is organized as follows. After an overall description of the sensor data acquisition and processing, the exercise classification technique is presented. In order to show how the proposed methodology could perform automatic fitness activity diarization, some results related to the recognition of simple exercises are reported and discussed. Finally, some conclusions end this work.

## 2. Materials and Methods

### 2.1. Data Acquisition and Preprocessing

**Figure 1:**
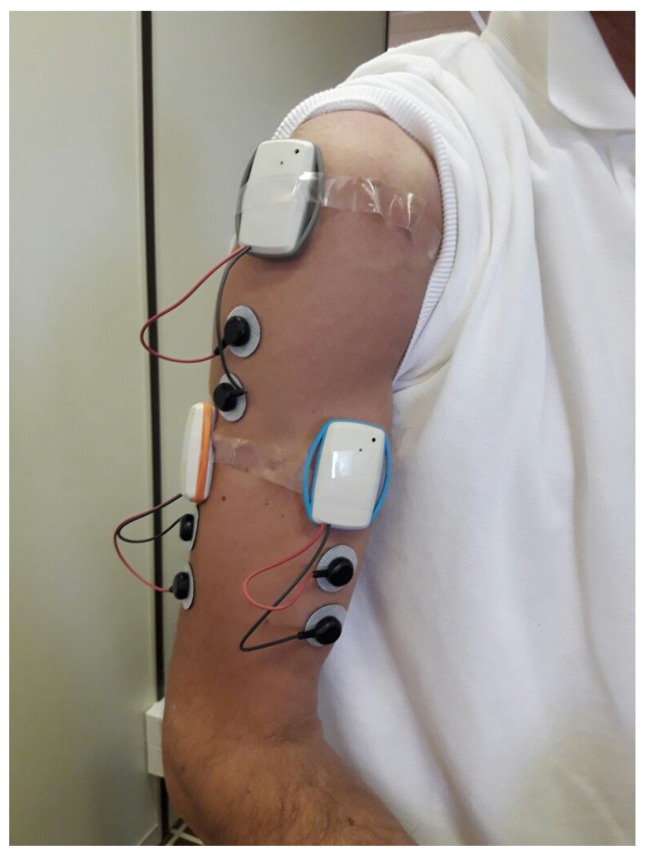
Recording setup. Photograph of the recording setup with the wireless electromyograph sensors worn on the upper right arm with their electrodes placed on the *biceps brachii*, *deltoideus medius*, and *triceps brachii* muscles.

The signals recorded for demonstrating the automatic exercise diarization system were obtained following the protocol outlined in [34,35]. In particular, a number of subjects, 10 in this study, worn three sets of sensors on their upper arm, as shown in Figure 1. It is expected that these three sets could eventually be integrated into a single stretch band so as to make their wearing easy and comfortable. Each set of sensors consists of a surface electromyogram acquisition unit coupled with a three-axis accelerometer [19].

The electrodes for sEMG acquisition were placed on the *biceps brachii*, *deltoideus medius*, and *triceps brachii* muscles following, as far as their location and orientation is concerned, common SENIAM [36] recommendations. The accelerometers where oriented so that their “Z” direction was perpendicular to the surface of the arm, pointing outwards, the “Y” direction parallel to the muscle, pointing downwards when the arm is at rest, and the “X” direction tangent to the surface to complete a right-handed frame. The placement of all the sensors on the upper arm, besides comfort and ease of application, also helps in limiting the effects of spurious movements of the hands and forearm on the acquired signals, especially on the acceleration ones, aiding in the classification of the particular exercises used in the experiment. Of course, a different set of exercises may require a different positioning of the sensors.

**Figure 2:**
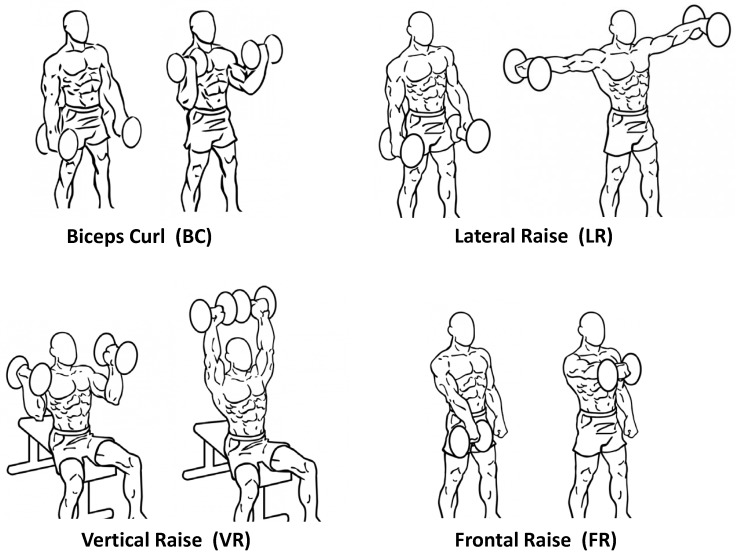
The four exercise types used in the experiments: starting and ending positions of each repetition. Source: http://db.everkinetic.com, licensed under the Creative Commons Attribution-Share Alike 4.0 International Public License.

The volunteering subjects were asked to perform sets of 10 to 12 repetitions of biceps curls, lateral raises, frontal raises, and vertical raises, as depicted in Figure 2. In between the lateral and frontal raises, an isometric contraction of the *biceps brachii* (essentially a biceps curl held still with the elbow at approximately 90°) was to be held for a few seconds. All the exercises were performed with either a 1 kg or a 3 kg dumbbell according to the subject’s own judgement about their fitness conditions, and all the participants gave their written informed consent in participating after having been instructed on the tasks to be performed.

A summary of the data obtained during this acquisition campaign is shown in Table 1, which reports the number of sets performed by each subject for each exercise type. After the recording session, start and end times of each type of exercise (a “segment”) were manually detected and labeled into the data files. The total duration of the collected data is reported in Table 2, together with the number of feature vectors extracted from the available segments. On average, over nine minutes of data is recorded for each type of exercise.

A small set of features was then extracted from the active portions of the recorded signals. For the purpose of feature extraction, sEMG and acceleration signals from the different nodes were considered independent.

The accelerometric signals, which were sampled at 125 Hz, were processed by first low-pass filtering them with a cut-off frequency of 0.625 Hz using an 8192 taps Blackman-Harris FIR filter with compensated group delay. Such a filtered signal is sliced into overlapping windows $a_{n}\left(t\right)$, each $T_{W}=$ 8 s long, shifted by approximately $T_{S}=$ 4 s (exact window shift ${\hat{T}}_{S}$ varies slightly in order to fit an integer number of whole windows within each manually segmented portion of the recording, i.e.,

(1) $${\hat{T}}_{S}=\left(T_{E}-T_{W}\right)/\left(\left\lceil T_{E}/T_{S}\right\rceil -1\right),$$

where $T_{E}$ is the duration of the segment). This procedure allowed the extraction of a total of 487 feature vectors.

For each window a “rotation vector” is estimated, based on the idea that the arm movement should be periodic, and the amount and axis of rotation is characteristic of the exercise being performed. To this end the extrema of the movement are located as the maxima of the function

(2) $$d_{n}\left(t\right)=∥a_{n}\left(t\right)-\bar{a_{n}\left(t\right)}∥$$

where $\bar{a_{n}\left(t\right)}$ denotes the time average of $a_{n}\left(t\right)$. If the movement is indeed periodic, it should be expected that these maxima are clustered into two distinct groups that correspond to the endpoints of the movement. Let’s call $c_{n}^{1}$, $c_{n}^{2}$ the averages of the maxima of $d_{n}\left(t\right)$ computed within these groups. The groups are numbered so that $c_{n}^{1}$ is the one corresponding to the rest condition, i.e., the one closest to the estimated acceleration due to gravity $\hat{g}$, which should be close to [0 −1 0] with the axes oriented as stated above. The direction of the rotation vector $r_{n}$ is then defined by the cross product between these two averages, and its modulo $\alpha_{n}$ is set to equal the angle between them, so as to represent the arm swing expressed in radians:

(3) $$r_{n}=\alpha_{n}\;\frac{c_{n}^{1}\times c_{n}^{2}}{∥c_{n}^{1}\times c_{n}^{2}∥}\;,$$

where $\alpha_{n}$ is the positive solution of

(4) $$\alpha_{n}=\arctan\frac{∥c_{n}^{1}\times c_{n}^{2}∥}{c_{n}^{1}\;\cdot \;c_{n}^{2}}\;.$$

The sEMG signal is preprocessed similarly. First, the sEMG, which is sampled at 2 kHz, is rectified and low-pass filtered using a 65536 tap Blackman-Harris FIR filter with compensated group delay and a cut-off frequency of 0.625 Hz just as with the acceleration filter.

This filtered signal, called mean absolute value (MAV), is normalized by dividing it by its average. This normalized signal will be sliced in overlapping windows $e_{n}\left(t\right)$, identical in duration and position to those used for the acceleration signals. As features, two simple propertied will be extracted for each window. Let $p_{n}$ be the mean of the peak values of the signal $e_{n}\left(t\right)$ within the window. We take as features both the averaged $m_{n}=\bar{e_{n}\left(t\right)}$ and the peak-to-mean ratio $z_{n}=p_{n}/m_{n}$.

**Figure 3:**
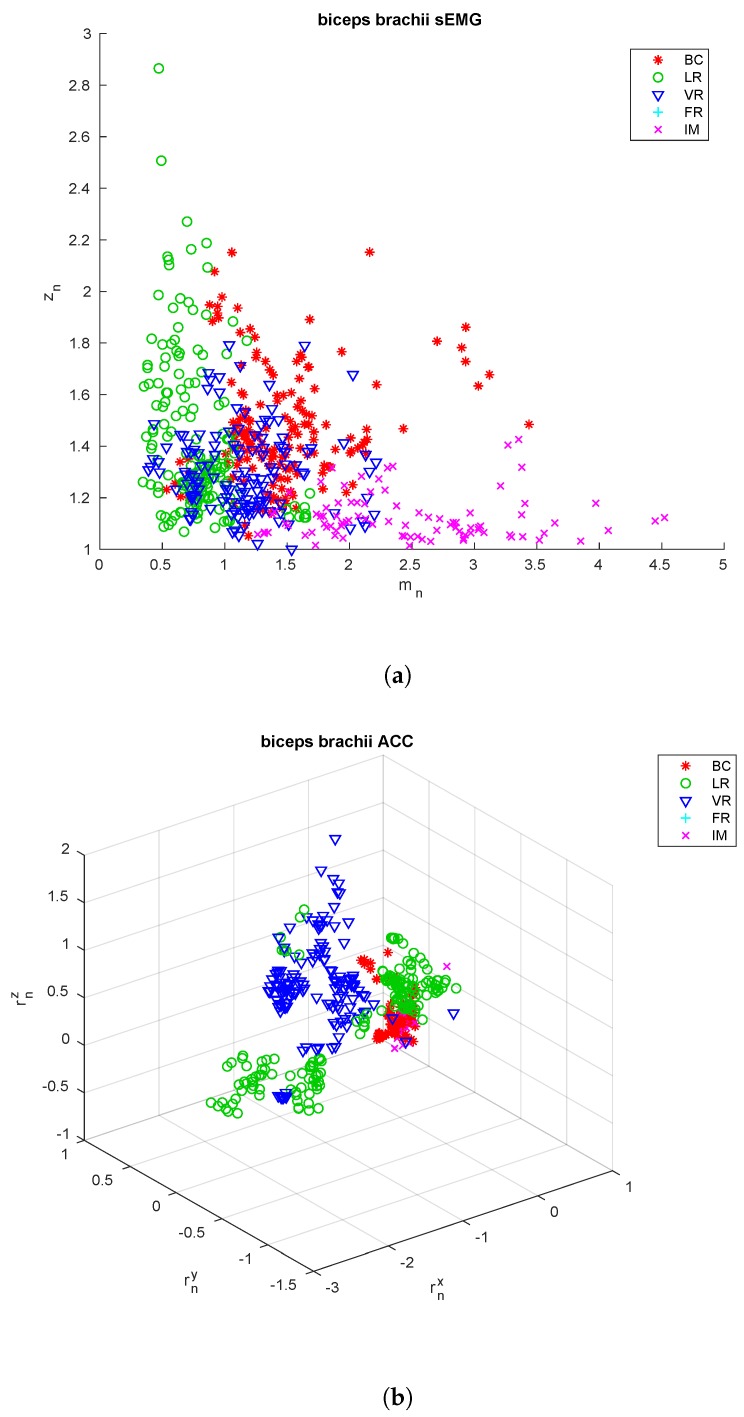
*Biceps brachii* features. Extracted from the sEMG signal (**a**) and the acceleration signals (**b**) from the sensors applied to the *biceps brachii* of all the subjects included in the training and testing sets.

**Figure 4:**
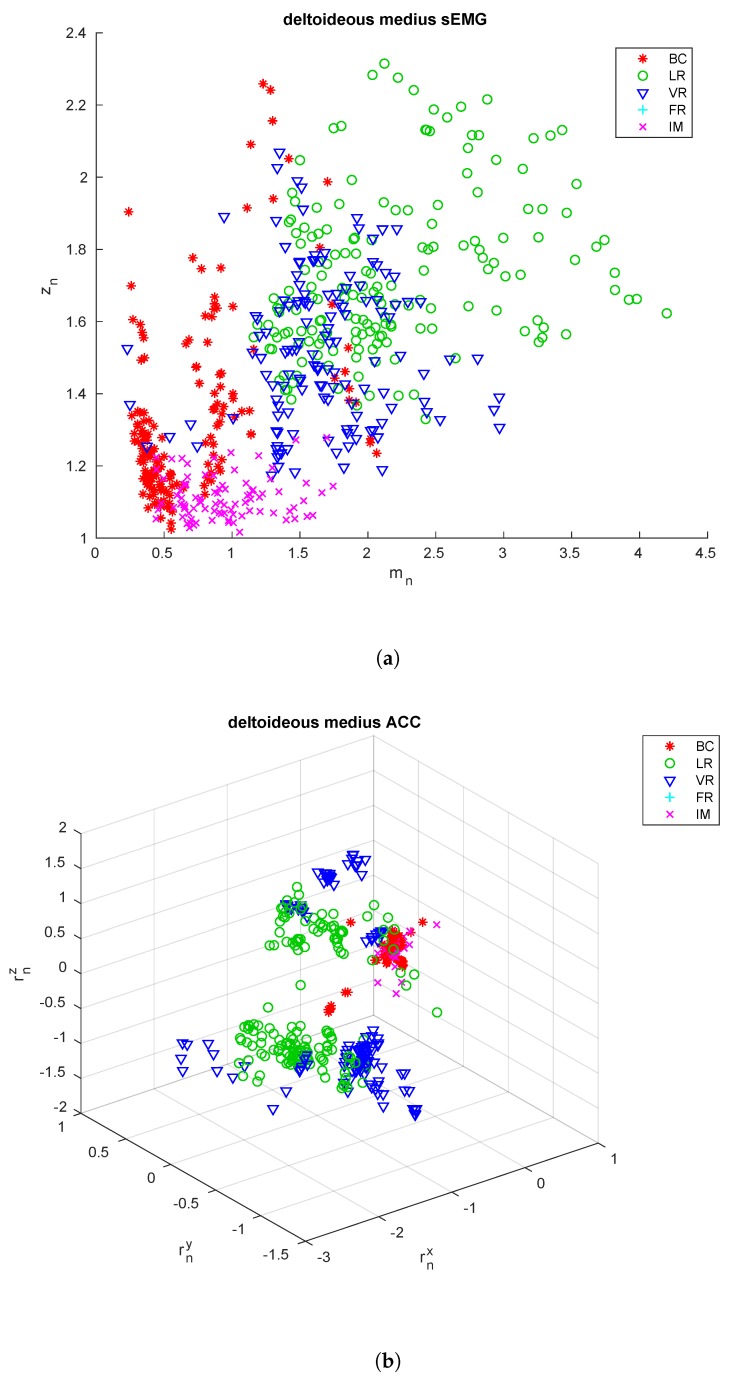
*Deltoideus medius* signals. Extracted from the sEMG signal (**a**) and the acceleration signals (**b**) from the sensors applied to the *deltoideus medius* of all the subjects included in the training and testing sets.

**Figure 5:**
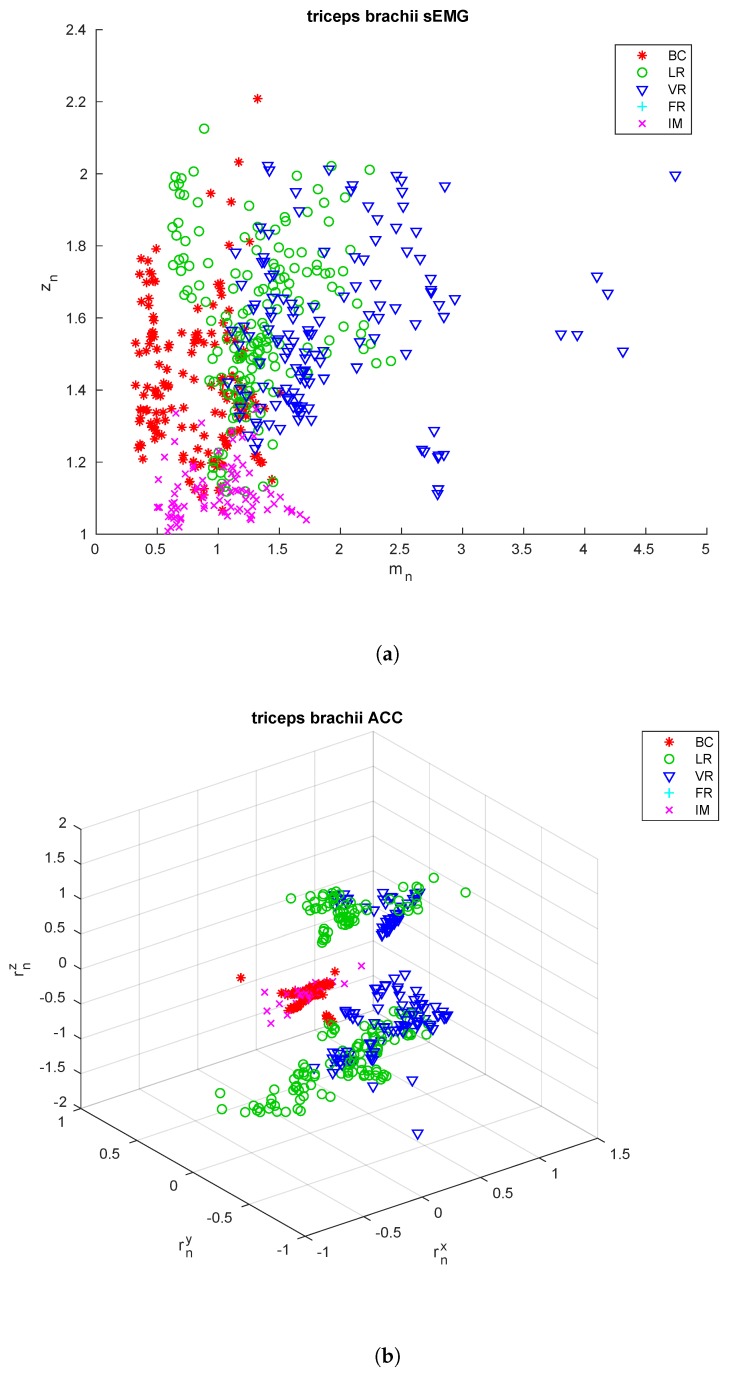
*Triceps brachii* signals. Extracted from the sEMG signal (**a**) and the acceleration signals (**b**) from the sensors applied to the *deltoideus medius* of all the subjects included in the training and testing sets.

The extracted features are shown in Figure 3, Figure 4 and Figure 5 for the three sensing nodes, respectively. From these feature it is already possible to see that the acceleration signals cannot be used alone to discriminate among all the exercise types, as e.g., the upper arm is essentially still during both biceps curls and isometric contractions, so the rotation vector is close to zero. On the other hand, these two exercises show very differing values for the parameter $z_{n}$, close to 1 for the isometric contraction, nearly double for biceps curls.

Due to some problems inherent in the sEMG measurements, including the separability and reproducibility of measurements, the size of discriminable arm exercices set was limited to 4 classes, excluding the frontal raises exercise from the rest of the analysis.

### 2.2. Classification

**Figure 6:**
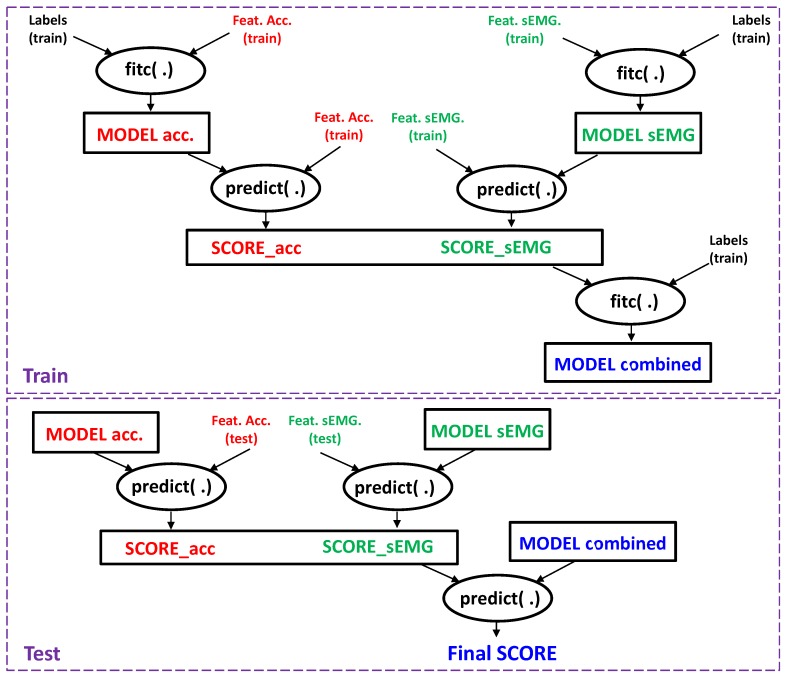
Classifier level fusion: flow of data and models.

When a classification task involves usage of different types of features, each of which has a different range of values due e.g., to different units of measure, it can be useful to perform a classifier-level fusion. It means that each feature set is used alone in a domain-specific classifier. The results of these classifiers are then combined to formulate the final response. A flow-chart of the process is outlined in Figure 6.

As can be seen, the two sets of features, i.e., those obtained by sEMG and by the accelerometers, are used separately to train two different recognizers (denoted by the “fitc” tag). The models so obtained are used to transform the original features into vectors of probabilities (“scores”), where each element contains the probability that the original feature vectors belongs to a certain class, by trying to recognize the training set again with the models just obtained (“predict”-tagged blocks).

The two score vectors obtained by the two first-level classifiers are then concatenated together (fused) to obtain the final feature vector, whose size is thus twice the number of classes. This will be used to train the final, second-level classifier to obtain the final model.

**Figure 7:**
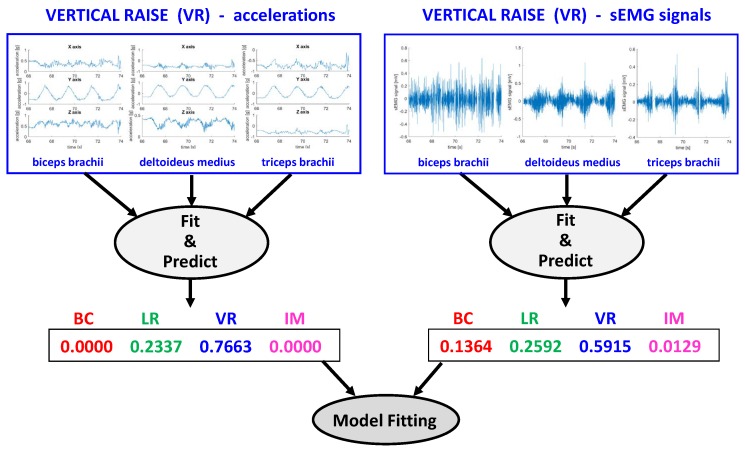
Example of the processing of a single signal frame through the training stage: time-domain signals (top) are used to train classification models, after feature extraction (not shown). Scores from these classification models are then fused together and used to train the final model.

An example of the process is shown in Figure 7, for the training phase. At the top of the figure, a single window of the signals corresponding to a portion of the vertical raise exercise is shown. Features are extracted from these signals and the acceleration-derived ones used (together with the rest of the training portion of the database) to train a first-stage classification model of the acceleration (left). The same is done for the sEMG-derived features (right). These very same features are then checked against the just trained models to obtain likelihood scores of the vector belonging to the various classes. In this example, acceleration alone could conclude that the signal was part of the vertical raise with 76.6% probability, while sEMG only with 59.2% probability, so fusing the results is straightforward. For more complicated cases, the role of the second-stage classifier is to help discriminate the correct class from the probabilities coming from the first stage. To this end, another classification model that maps the correct class to these probability vectors is finally trained.

When an unknown feature vector set arrive to be classified, it suffices to first transform it into score vectors using the previously trained first-level classifiers, and then feed the concatenated score vectors into the second-level classifier, as shown in the bottom panel of Figure 6. Of course, different choices of classifiers are possible at the various stages, as each one must deal with data of different kinds. In this work we limited our experimentation to the same classifier type for the two used in the first stage, and a different type for the second stage.

### 2.3. Results

To evaluate the effectiveness of this method, the acquired data was split into a training set and a testing set. Data from eight subjects (80% of the total number) was used as train material, while the remaining two subjects provided the testing material. Due to the limited number of subjects, all the 45 possible combinations of training/testing splits were tested, and the results averaged by adding the resulting confusion matrices.

Different readily-available classifiers have been tried at the first stage and at the second stage, namely support vector machines (SVM) [37] with polynomial (SVMp), Gaussian (SVMg), and linear (SVMl) kernels, decision trees (tree) [38], *k*-nearest neighbours classifiers (KNN) [39], and linear discriminant analysis (LDA) [40].

A summary of the resulting accuracy is shown in Table 3, which reports, for each first-stage classifier type (rows), its accuracy when used alone on a single set of features, and the overall accuracy when combined with a possibly different type of second-level classifier. The data reported in the table show the average accuracy, together with the minimum and maximum accuracies achieved by each pair of recognizers across all the possible train/test splits. These latter two results help in comparing the consistency of the 36 different recognition algorithm pairs.

As can be seen, using the correct combination of classifiers, this fusion technique can improve the overall accuracy with respect to using only one type of feature.

Table 4 reports confusion matrices resulting from the best combinations of recognizers. Each row shows the classification results for each kind of exercise, where BC stands for biceps curls, LR for lateral raises, VR for vertical raises, and IM for the isometric contraction. The column labels represent the estimated exercise types. The best was chosen both in terms of average accuracy (SVMl/LDA) and in terms of highest consistency, i.e., highest worst-case accuracy (KNN/SVMp). There is little difference between these two combinations in terms of average performance, but the KNN/SVMp combination achieved a much better worst-case accuracy, and can thus be considered to be better suited to handle well most situations.

## 3. Conclusions

A classifier level data fusion approach for accelerometer and sEMG signals, acquired from a wearable wireless system, was presented. The wireless system consists of three sets of devices each of them being a sEMG acquisition sensor coupled with a three-axis accelerometer. It is aimed at monitoring human activity during sports and fitness activities, to provide an automatic diarization of the exercises performed. To demonstrate this capability of the system data recorded from several subjects (wearing the sensors on their upper arm and performing different physical exercises) were used to train and test the two-level automatic classifier for recognizing the type of exercise being performed, achieving an overall accuracy of 82.6% over four different types of activities.

## Figures and Tables

**Table 1:** Database consistency: number of sets performed by each subject for each exercise type, with the specified dumbbell weight.

Subject	Gender	Weight	BC	LR	VR	FR	IM
1	M	3 kg	2	2	2	2	2
2	M	3 kg	2	2	2	2	3
3	M	3 kg	2	2	1	0	1
4	M	3 kg	1	2	2	2	1
5	M	3 kg	2	2	2	2	2
6	M	3 kg	1	1	1	1	1
7	M	3 kg	2	2	2	2	2
8	M	3 kg	3	3	2	3	3
9	F	1 kg	2	2	2	2	2
10	M	3 kg	2	2	2	2	2

**Table 2:** Database consistency: recorded duration for each exercise type and subject [seconds (number of feature vectors)].

Subject	BC	LR	VR	FR	IM
1	67 (18)	74 (20)	65 (17)	68 (18)	50 (13)
2	54 (14)	51 (13)	36 (10)	47 (13)	40 (11)
3	74 (19)	72 (19)	35 ( 9)	0 ( 0)	3 ( 0)
4	41 (11)	65 (17)	62 (16)	49 (13)	37 ( 9)
5	69 (18)	61 (16)	59 (16)	71 (19)	28 (08)
6	32 ( 8)	28 ( 7)	29 ( 8)	27 ( 7)	23 ( 6)
7	87 (23)	99 (26)	71 (18)	83 (22)	65 (17)
8	90 (23)	93 (24)	56 (15)	96 (24)	51 (13)
9	52 (14)	59 (16)	51 (14)	53 (14)	25 ( 7)
10	59 (15)	60 (16)	57 (15)	58 (15)	24 ( 6)
Total	625 (163)	662 (174)	521 (138)	552 (145)	346 (90)

**Table 3:** Accuracy from the 2-stage classifier level fusion for different classifiers employed at the two stages, compared with the accuracy attainable without fusion using the same classifiers and only one set of features at a time. Reported figures are average percentages (top), together with minimum and maximum accuracies (bottom) achieved across all the possible 80%/20% train/test splits.

1st Stage		No Fusion			2nd Stage Classifier					
		ACC	EMG		SVMp	SVMg	SVMl	Tree	KNN	LDA
**SVMp**		73.5 48.1–96.0	75.3 43.9–99.2		80.3 57.5–98.0	61.2 41.5–78.7	80.6 57.5–98.0	70.0 45.0–96.0	71.4 46.3–87.5	82.1 59.4–100
**SVMg**		59.6 40.2–73.8	48.3 32.1–61.1		53.8 36.5–68.3	28.1 16.8–35.5	47.5 31.0–71.3	39.5 20.6–63.0	63.9 49.1–78.7	36.3 8.6–49.7
**SVMl**		64.3 41.2–93.9	81.4 49.5–99.0		79.1 43.9–99.2	58.5 38.1–70.3	81.8 49.0–100	78.5 45.5–99.2	79.6 45.9–100	82.6 50.0–100
**Tree**		62.2 36.2–89.3	71.9 42.9–91.2		66.0 37.8–96.9	63.3 39.4–83.5	65.8 39.4–96.9	65.4 43.2–86.9	66.1 40.4–83.6	64.4 38.7–92.1
**KNN**		75.1 42.5–91.8	74.6 45.9–96.1		80.6 63.2–97.5	77.6 48.0–93.0	75.9 46.2–90.8	75.1 42.5–91.8	79.9 59.8–97.5	30.8 26.0–36.0
**LDA**		66.6 40.7–88.0	77.7 49.5–100		76.5 48.4–99.0	74.1 40.8–97.1	80.2 51.0–99.2	77.6 50.5–98.8	78.4 59.7–98.4	80.4 52.5–99.2

**Table 4:** Average confusion matrix resulting from the SVMl/LDA classifier combo, which achieved the best overall accuracy of 82.6% (left), and from the KNN/SVMp classifier combo, which achieved the most consistent accuracy ranging from 63.2% to 97.5%, with an average of 80.6% (right).

		BC	LR	VR	IM			BC	LR	VR	IM	
	**BC**	143.6	0.4	6.6	12.4		**BC**	146.4	4.1	4.0	8.4	
	**LR**	1.0	140.2	32.8	0.0		**LR**	0.0	171.8	2.2	0.0	
	**VR**	1.0	36.1	100.9	0.0		**VR**	3.3	67.1	67.6	0.0	
	**IM**	8.0	0.0	0.0	82.0		**IM**	20.1	0.0	0.0	69.9	
